# Enzymatically Triggered Drug Release from Microgels Controlled by Glucose Concentration

**DOI:** 10.1021/acsbiomaterials.4c01721

**Published:** 2024-10-02

**Authors:** Klaudia Kaniewska, Marcin Mackiewicz, Oleh Smutok, Mykhailo Gonchar, Evgeny Katz, Marcin Karbarz

**Affiliations:** † Faculty of Chemistry, University of Warsaw, 1 Pasteura, Warsaw, PL 02-093, Poland; ‡ Biological and Chemical Research Center, University of Warsaw, 101 Żwirki i Wigury Av., Warsaw, PL 02-089, Poland; § Department of Chemistry and Biomolecular Science, Clarkson University, Potsdam 13699, New York, United States; ∥ Institute of Cell Biology, National Academy of Sciences of Ukraine, Lviv 79005, Ukraine

**Keywords:** smart microgels, drug release, enzyme, pH-sensitive system, glucose

## Abstract

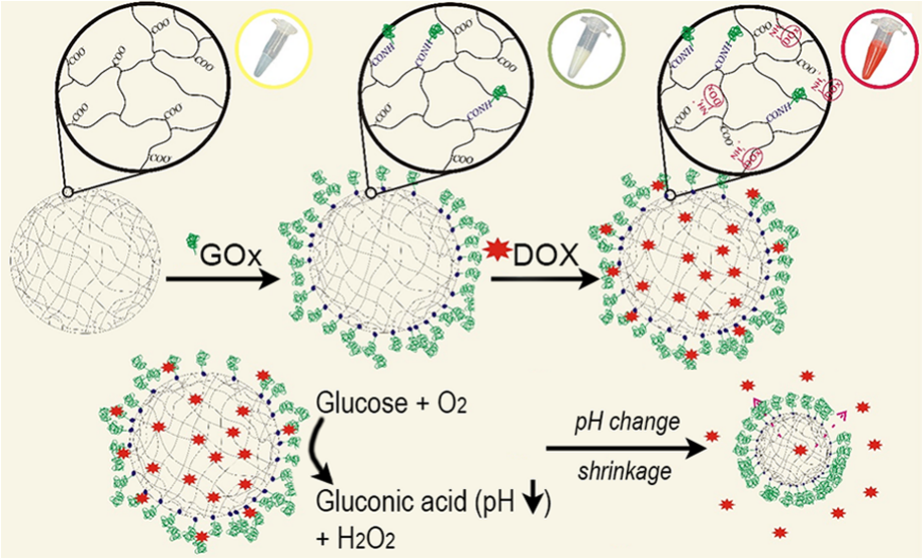

This study aims to design microgels for controlled drug release via enzymatically generated pH changes in the presence of glucose. Modern medicine is focused on developing smart delivery systems with controlled release capabilities. In response to this demand, we present the synthesis, characterization, and enzymatically triggered drug release behavior of microgels based on poly(acrylic acid) modified with glucose oxidase (GOx) (p(AA-BIS)-GOx). TEM images revealed that the sizes of air-dried p(AA-BIS)-GOx microgels were approximately 130 nm. DLS measurements showed glucose-triggered microgel size changes upon glucose addition, which depended on buffer concentration. Enzymatically triggered drug release experiments using doxorubicin-loaded microgels with immobilized GOx demonstrated that drug release is strongly dependent on glucose and buffer concentration. The highest differences in release triggered by 5 and 25 mM glucose were observed in HEPES buffer at concentrations of 3 and 9 mM. Under these conditions, 80 and 52% of DOX were released with 25 mM glucose, while 47 and 28% of DOX were released with 5 mM glucose. The interstitial glucose concentration in a tumor ranges from ∼15 to 50 mM. Normal fasting blood glucose levels are about 5.5 mM, and postprandial (2 h after a meal) glucose levels should be less than 7.8 mM. The obtained results highlight the microgel’s potential for drug delivery using the enhanced permeability and retention (EPR) effect, where drug release is controlled by enzymatically generated pH changes in response to elevated glucose concentrations.

## 1 Introduction

In recent years, a strong emphasis has been focused on the development of drug delivery systems (DDS).^1−4^ This important medical technology helps to minimize side effects in drug treatments simultaneously increasing its efficacy. The materials used as DDS have to meet specific requirements: they should be monodispersed, and protect the medicine from degradation or undergo undesired metabolism, and be biocompatible. More advanced “smart” systems can provide features such as controlled drug release on demand, targeted delivery, and the ability to degrade over time or to release drugs instantly or over a prolonged period.^5−8^

Various materials have been studied for use as DDS, ranging from inorganic materials like metal nanoparticles, quantum nanodots, and MOFS to organic material liposomes, micelles, and micronanogels.^9−15^ Hydrogels, a class of materials extensively investigated in terms of drug delivery systems, can possess the necessary properties mentioned above. The structure of polymer network can be adjusted according to the needs, incorporating both positive or negative charges, as well as being sensitive to different triggers.^16,17^ Their polymer network can be also modified with targeting molecules. Hydrogel delivery systems can be obtained in sizes ranging from nano- to macro-,^18−20^ achieving the desirable size for the enhanced permeability and retention (EPR) effect, which improves the accumulation of particles in tumor tissues.^21^ Additionally, nano- and microgels can be made degradable^22,23^ and can modify electrode surfaces.^24,25^ Marcisz et al. presented a biosensor based on an electrode covered with thermosensitive microgels. These microgels act as electroactive mediating biosystems due to the covalent bonding of glucose oxidase and ferrocene.^26^

The ability to design hydrogels with controlled properties makes them the preferred materials for DDS. The starting point in designing a new delivery system is to answer what will be delivered and where. The specific conditions at the target site determine the properties that the material should have. In our case, we targeted some tumor cells and chose doxorubicin (DOX) as the model drug. DOX has an amine group with p*K*_b_ = 9.9, and in neutral and acidic pH, it is in the protonated form, which is well soluble in water.^27^

The cancer environment differs from the healthy one. For instance, while blood pH is around 7.4, whereas the extracellular pH in tumor tissues ranges from 6.5 to 7.2 and further decreases to 5.0–5.5 in endosomes and lysosomes.^28^ This property has been utilized, for instance, by Licea-Navarro et al., who developed PEGylated nanogels for the delivery of the anticancer drug 5-fluorouracil to explore its potential applications in colon cancer therapies. The drug was released faster at pH 5 than at pH 7.4.^29^ In the cancer environment, there is usually a higher concentration of hydrogen peroxide, which can be used for the decomposition of drug carriers and subsequent drug release.^23,30^ For example, Yan et al. obtained hydrogen peroxide-responsive nanocarriers containing selenide groups. The oxidation of these selenide groups resulted in the rapid disassembly of the nanocarriers and the release of the drug.^31^

Cancer cells absorb glucose at a rate that is 20–30 times greater than normal cells, as their growth relies significantly on glucose metabolism for energy. Despite this rapid uptake, the interstitial glucose concentration in tumors remains high, ranging from approximately 15 to 50 mM. This level fluctuates depending on the cancer cell type and growth rate. Under hyperglycemic conditions, glucose levels in tumors can rise by 13–17 times.^32^ In contrast, the normal glucose range for fasting blood level is about 5.5 mM, and postprandial (2 h after a meal) glucose levels should be less than 7.8 mM. On the other hand, in diabetics, fasting blood glucose levels exceed up to 11 mM. Glucose-sensitive hydrogels exhibit different responses depending on the glucose levels present. These hydrogels are valuable in creating self-regulating delivery systems that can administer the required insulin dose in accordance with blood glucose concentrations. This functionality can be achieved by integrating hydrogels loaded with glucose oxidase, lectins, or phenylboronic acid groups.^33^

The aim of this work was to achieve a new anticancer carrier for a “smart” drug release system that utilizes differences in glucose concentration between the blood and tumor environment, where the release of medicine is triggered by an enzymatically generated pH change. The carrier in the form of microgels is based on acrylic acid (pAA-BIS) with covalently immobilized enzyme (glucose oxidase, GOx) and electrostatically attached drug (through interaction between the carboxylic groups of the microgel and the amine groups of doxorubicin). The proposed novel system is a specific microreactor where the presence of the target enzymatic substrate (glucose), the GOx produces gluconic acid. The local generation of acid causes a decrease in pH, which consequently leads to the release efficacy of doxorubicin. We believe that this concept can be extended to other enzymes and target substrates.

## 2 Materials and Methods

### 2.1 Materials

Acrylic acid (AA), *N,N’*–methylenebis(acrylamide) (BIS), azobis(isobutyronitrile) (AIBN), glucose oxidase (GOx from *Aspergillus niger*, 158,523 U/mg), *N*-hydroxysuccinimide (NHS), *N*-(3-(dimethylamino)propyl)-*N*′-ethylcarbodiimide (EDC), 2-[4-(2-hydroxyethyl)piperazin-1-yl]ethanesulfonic acid (HEPES), glucose, horseradish peroxidase (HRP, 279 U/mg), 2,2′-azino-bis(3-ethylbenzothiazoline-6-sulfonic acid) (ABTS) were purchased from Sigam-Aldrich. Doxorubicin (DOX) was obtained from ABCR. Acetonitrile, sodium hydroxide, and chloric acid were purchased from POCh. All chemicals were used as received. All solutions were prepared using high-purity water obtained from a Milli-Q Plus/Millipore purification system (water conductivity: 0.056 μS/cm).

### 2.2 Dynamic Light Scattering

A Malvern Zetasizer instrument (Nano ZS, UK) equipped with a 4 mW Helium–Neon laser with a light wavelength of 632.8 nm and scattering angle of 173° was used to determine the following quantities: scattered light intensity of the p(AA-BIS) microgel, hydrodynamic diameter and polydispersity index (PDI). The ζ potential was measured using the same instrument. A folded capillary cell with gold electrodes was employed for this purpose. The analyzer calculated the ζ potential from the electrophoretic mobility using the Henry equation and the Smoluchowski approximation.

### 2.3 Scanning and Transmission Electron Microscopy (SEM and TEM)

The SEM images were taken with a Zeiss Merlin field-emission instrument. Before the imagining, the samples were coated with a 3 nm thick layer of sputtered Au–Pd alloy using a Polaron SC7620 mini sputter coater. The gel samples for TEM were prepared by placing a drop of an aqueous suspension of the tested microgel and microcomposite particles on a Formvar-coated copper grid and allowing them to dry in the air. Samples were inspected using a TALOS F200X microscope (Thermo Scientific).

### 2.4 UV–vis Measurements

The release profiles were registered with an Evolution 300 UV–vis spectrophotometer Thermo Scientific. The spectra were collected after a certain amount of time in the wavelength range of 400–700 nm.

## 3 Preparation of the p(AA-BIS)-GOx-DOX Microgel

### 3.1 Synthesis of the p(AA-BIS) Microgel

**Scheme 1:**
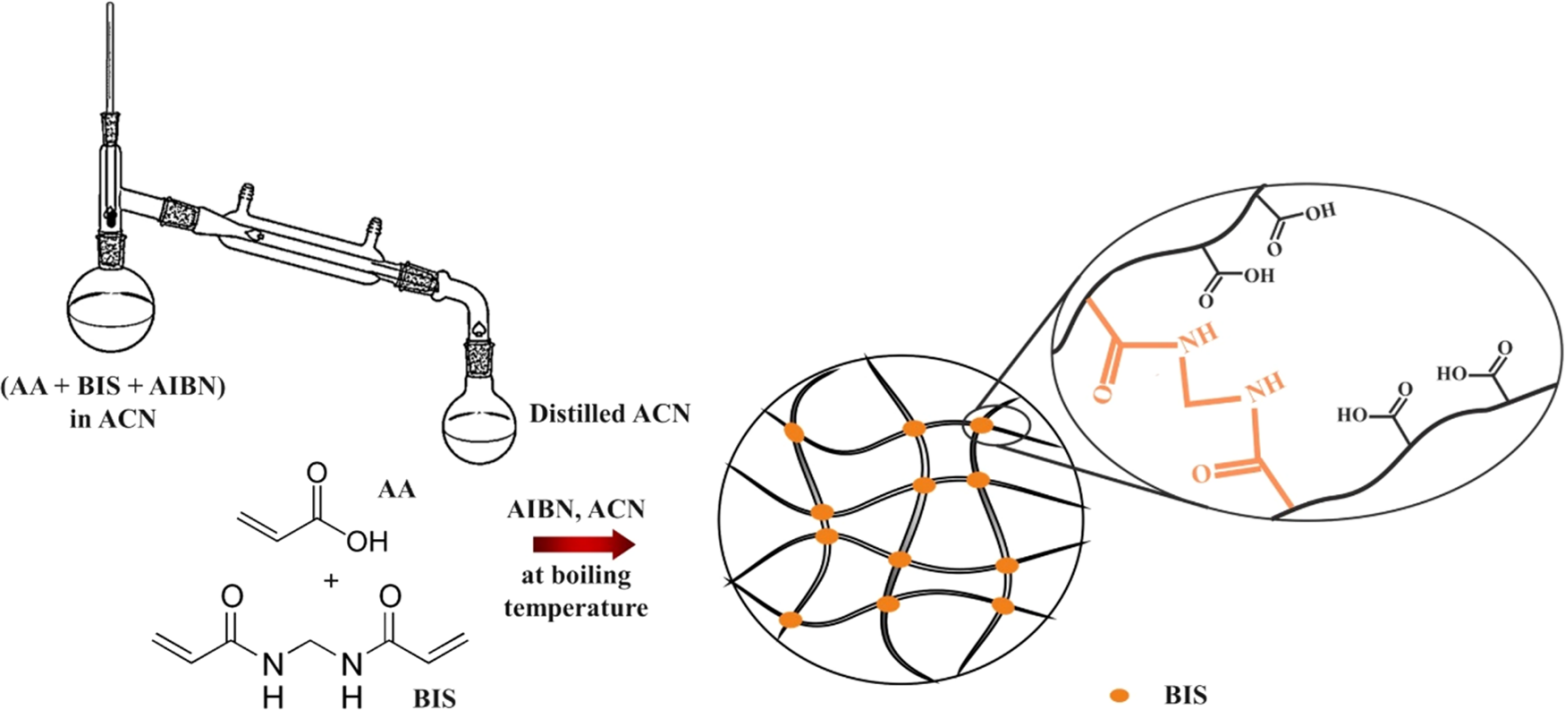
Scheme of Synthesis of p(AA-BIS) Microgels Carried Out by Means of Distillation Polymerization

The p(AA-BIS) microgels were synthesized using a modified procedure described previously.^3^ In this approach, distillation polymerization was employed. Specifically, AA (1.55 mL), BIS (0.1063 g, 3 mol %), and AIBN (0.3620 g) were dissolved in 78 mL of ACN and placed in a reactor connected to a fractionating column with a Liebig condenser and a receiver (Scheme 1). The reaction proceeded at the boiling point of ACN until 30 mL of acetonitrile was collected in the receiver. The resulting product was purified through repeated centrifugation and redispersion in water.

### 3.2 Functionalization of p(AA-BIS) with an Enzyme

The p(AA-BIS) microgels were then modified with the enzyme (GOx) using an EDC/NHS coupling approach. The acetonitrile was replaced with water by centrifuging the microgel solution four times. After the final centrifugation, the supernatant was replaced with a fresh enzyme solution (100 U/mL). After 1 h, EDC and NHS were added at concentrations of 100 mM each. The coupling reaction was carried out overnight, which is sufficient time for the reaction to be completed.^34^ The next day, the microgel was centrifuged eight times, and the supernatant, containing residues such as enzymes unattached to the microgel, was replaced with fresh deionized water. The dispersion of the p(AA-BIS)-GOx microgel was concentrated twice during the last centrifugation. The final concentration of the p(AA-BIS)-GOx microgel was determined by weighing the dry mass from 1 mL of the microgel solution, resulting in a concentration of 4.5 mg/mL.

### 3.3 Drug Loading

The solution of the model drug, doxorubicin (DOX), with pH adjusted to 7 was added to p(AA-BIS)-GOx microgel solution (such that 0.5 mL of DOX was added to 1 mL microgel solution). The final concentration of DOX was 0.5 mg/mL. The loading process lasted overnight. The final microgel p(AA-BIS)-GOx-DOX was obtained after purification from unbound DOX by dialysis in a 10 kDa membrane.

After 5 h of purification the water after dialysis was investigated using UV–vis, and it was found that the loading efficiency, as defined by eq 1, was very close to 100%.
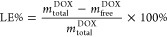
1

The drug loading capacity (DLC) was determined using eq 2
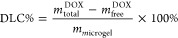
2where *m*_total_^DOX^ is the total mass of DOX used in the loading process, *m*_free_^DOX^ is the mass of DOX that was not bound to the microgel which is equal to 0, and *m*_microgel_ is the mass of the dry microgel which is 3.4 mg per 1 mL. The determined DLC equaled approximately 15%.

### 3.4 Determination of Enzymatic Activity of p(AA-BIS)-GOx-DOX Microgels

The activity of GOx immobilized in p(AA-BIS)-GOx-DOX microgel was measured by performing a standard enzymatic assay of GOx in the presence of HRP and ABTS.^35,36^ The enzyme activity was determined from a calibration curve obtained spectrophotometrically at 415 nm, corresponding to the maximum absorbance of ABTS. The calibration curve was plotted as the slope of the absorption of oxidized ABTS over time versus enzyme activity. The reactant solution included: 3 mM HEPES buffer, pH 7, varying concentrations of GOx in the range of 2 to 0.005 U/mL, 2 mM glucose, 5 U/mL HRP, 0.1 mM ABTS, and O_2_ at equilibrium with air. To determine the activity of the modified microgel, 100 μL of p(AA-BIS)-GOx-DOX microgel was added to the reactant solution (3 mM HEPES buffer, pH 7, 2 mM glucose, 5 U/mL HRP, 0.1 mM ABTS, and O_2_ at equilibrium with air) and measured. The total volume was 1 mL. The final result takes into account a 10-fold dilution.

### 3.5 Payload Release Investigation

**Figure 1:**
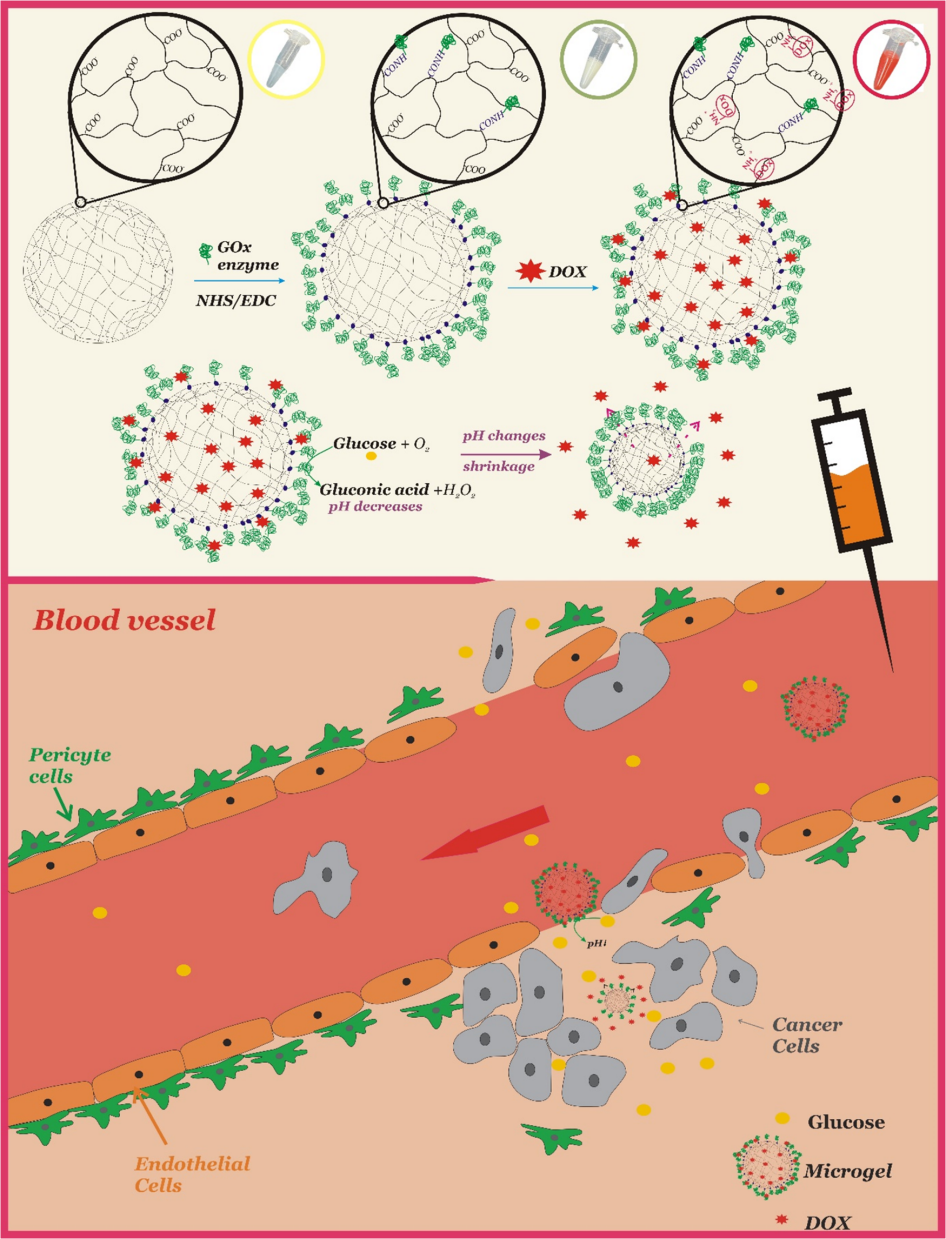
Scheme of functionalization of microgel p(AA-BIS) with GOx and loading of DOX (above), and its potential operation in tumor environment (below).

One milliliter of hydrogel was poured into the dialysis’s membrane with 10 kDa pore size and kept in 10 mL of HEPES buffer solution of pH = 7 at different concentrations (0, 3, 6, 9, and 30 mM). First, the leakage was checked for 1 h, after that time the glucose was added in two concentrations 5 and 25 mM (for each HEPES concentration), and as a control, the sample without added glucose was measured. The release profile was presented as a percentage of the released drug, calculated via its absorbance at 476 nm (corresponding to DOX maximum absorbance) at specific times normalized against the absorbance of the total drug concentration over time. The absorbance representing the total released drug from the microgels was determined for each sample after a 48 h release period. This determination was achieved by acidifying the solution to pH 2.8 using 1 M hydrochloric acid. pH 2.8 is more than two units below the p*K*_a_ of poly(acrylic acid), leading to the protonation of nearly all carboxylic groups and the disappearance of electrostatic interactions between the matrix and the drug. Under such conditions, 100% of the drug is expected to be released.^37^ The schematic illustration of the modification and release of DOX from the microgel is shown in Figure 1.

## 4 Results and Discussion

### 4.1 Characterization of the Dry Microgel

**Figure 2:**
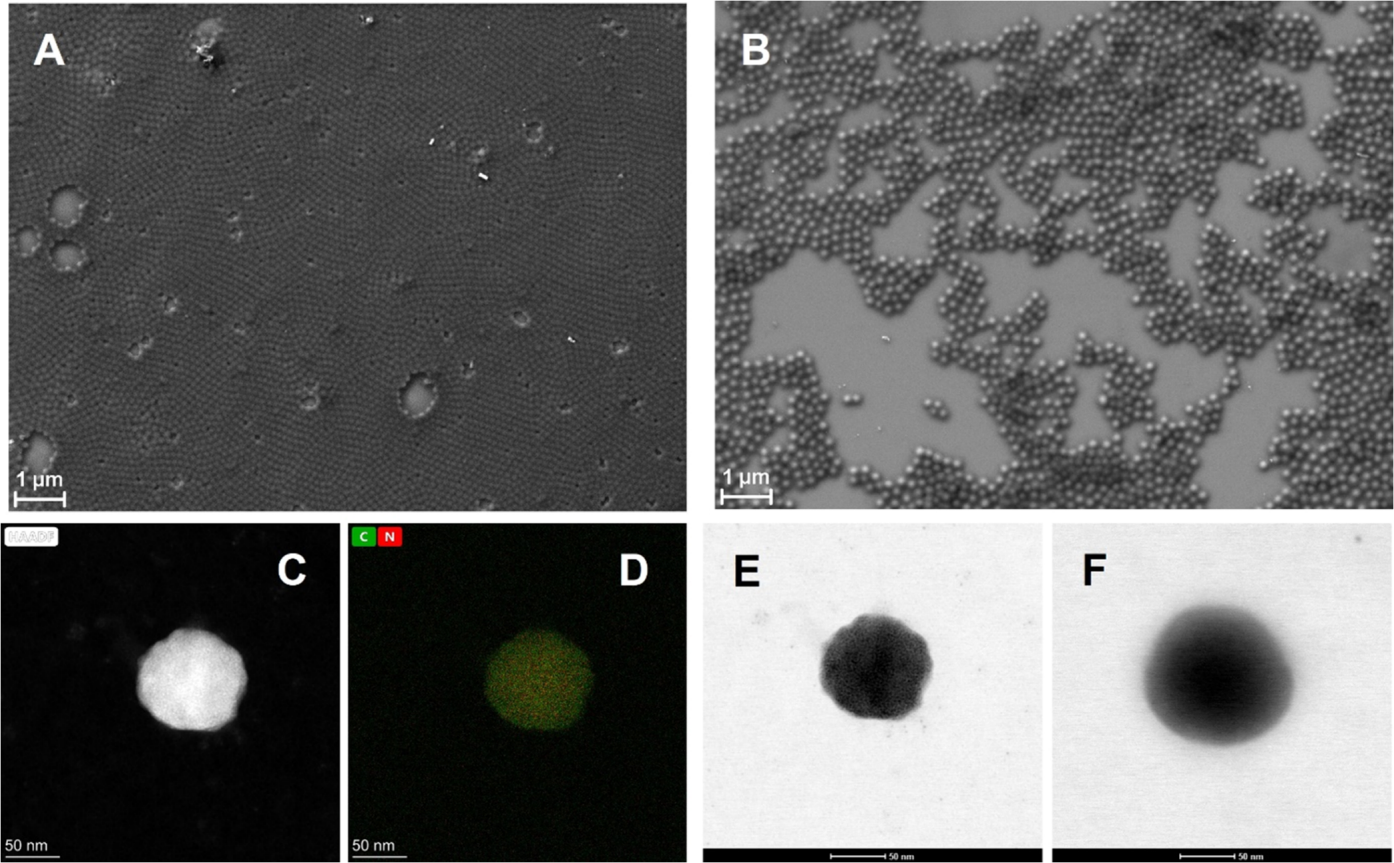
SEM microphotographs of air-dried (A) p(AA-BIS) microgel, (B) p(AA-BIS)-GOx microgel, (C) HAADF and (D) EDS-HAADF elemental mapping of carbon and nitrogen. TEM images of (E) p(AA-BIS) microgel and (F) p(AA-BIS)-GOx microgel.

The size and morphology of synthesized gel particles were visualized using scanning and transmission electron microscopy (Figure 2). The determined diameter of air-dried p(AA-BIS) microgels, based on the TEM images, was approximately 100 nm. However, the p(AA-BIS)-GOx microgels were larger, with a size of approximately 130 nm. The increase in size after modification with the enzyme is probably due to the formation of a layer of self-cross-linked enzyme particles on the surface of the microgels. In both cases, SEM images show particles with a smooth surface and spherical shape, with individual microgels having a uniform size distribution clearly visible both before and after enzyme modification (see Figure 2A,B). Complementary TEM images show that microgels in the dry state before enzyme modification are more compact and have a wavy surface, whereas after modification, the microgels have a looser external layer of self-cross-linked enzyme particles (Figure 2E,F). High-angle annular dark-field (HAADF) and EDS-HAADF images of microgel particles before modification indicate that the cross-linker is evenly distributed throughout the entire volume; nitrogen originates from BIS, whereas carbon comes from both monomers (Figure 2C,D).

Microgel samples, both before and after enzyme modification, were analyzed using EDS-HAADF methods for a quantitative examination of key elements (C, N, and O). The EDS spectra presented in Figure 1S clearly show a significant increase in nitrogen content after modification. Additionally, the elemental mapping of nitrogen (Figure 1BS) reveals that nitrogen is primarily concentrated in the outer part of the microgel particles, indicating the formation of a self-cross-linked enzyme layer on the microgel surface. This is attributed to the significantly higher nitrogen content in the enzyme compared to the polymer network, where nitrogen originates only from the cross-linker.

Microgels, both before and after modification with the enzyme, were also investigated using FTIR spectroscopy. After the modification, the characteristic signals corresponding to the peptide bond are clearly observed (Figure 2S). These include a broad signal located at 3310 cm^–1^, characteristic of the stretching of N–H bonds in amide groups, as well as the Amide I and Amide II bands located at 1620 and 1540 cm^–1^, respectively. This is also evidence of the successful modification of the microgel with the enzyme.

### 4.2 Characterization of the Swollen Microgel

**Figure 3:**
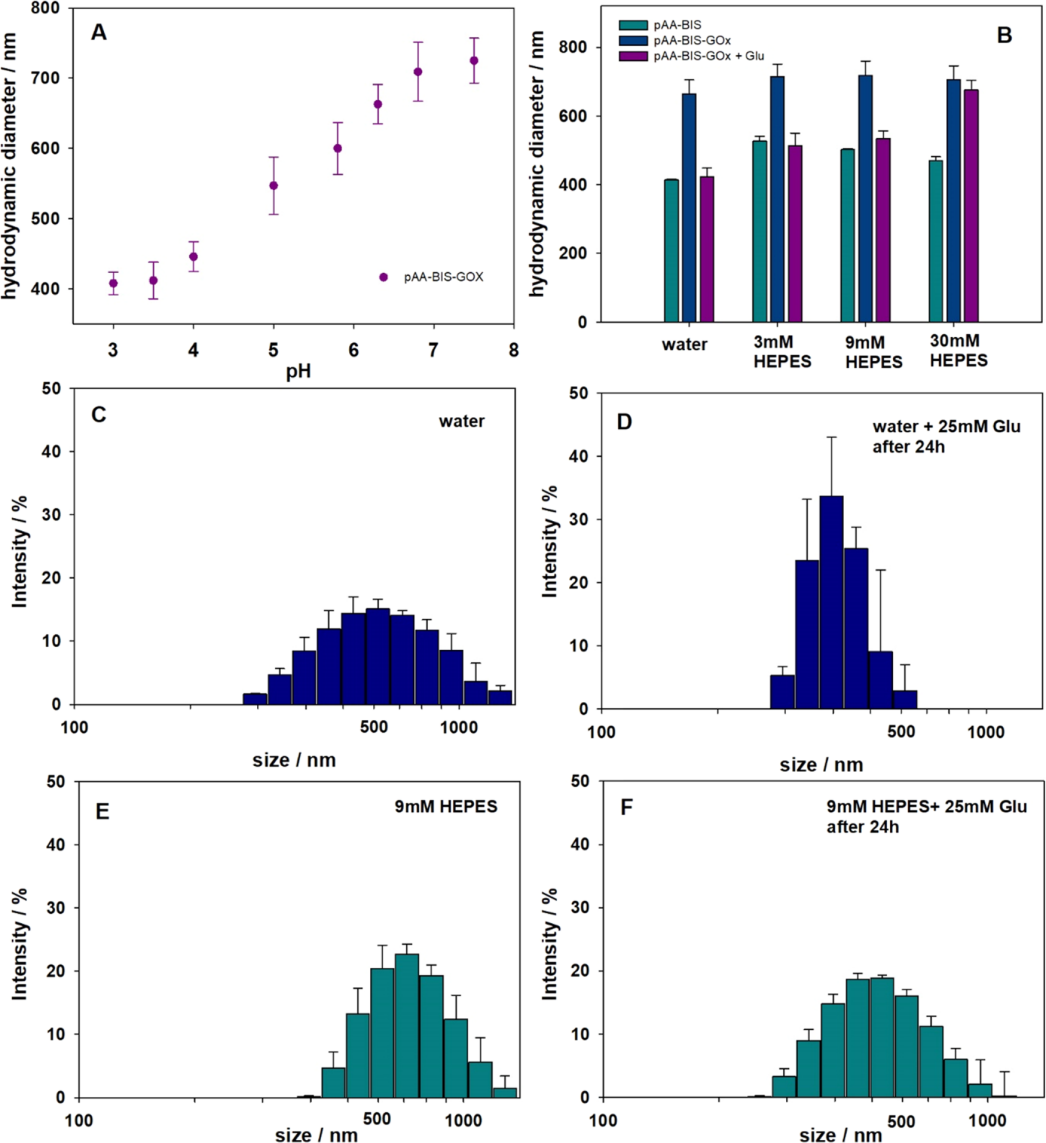
Size presented as the hydrodynamic diameter of p(AA-BIS)-GOx microgel as a function of pH (A), size of p(AA-BIS) and p(AA-BIS)-GOx microgels before and after addition of glucose (Glu) in water and HEPES buffer with 3, 9, and 25 mM, respectively (B). The distribution of p(AA-BIS)-GOx microgel size presented by intensity in water without glucose addition (C), and in water with 25 mM glucose added after 24 h (D), in 9 mM HEPES without glucose (E), and with 25 mM of glucose after 24 h (F).

Further microgel (microbeads) characterization was carried out in an aqueous environment. First, the swollen microgel size was determined in water at different pH levels, and adjusted by adding hydrochloric acid to p(AA-BIS)-GOx (Figure 3A). The hydrodynamic diameter (*d*_h_) of p(AA-BIS)-GOx decreases with a drop in pH, similar to unmodified microgels based on poly(acrylic acid).^38,39^ The chemical immobilization of the enzyme through the formation of an amide bond between the amine groups of glucose oxidase did not affect the internal structure of the microgels. Therefore, the modified p(AA-BIS)-GOx still exhibits sensitivity to pH changes. Acidification of the environment leads to protonation of carboxylic groups, a drop in osmotic pressure, and the formation of hydrogen interactions between COOH groups, which is observed as shrinkage of the microgels. Next, DLS measurements were used to determine the size of hydrated microgels before and after modification with the enzyme, as well as the effect of glucose presence on the size of p(AA-BIS)-GOx microparticles under different conditions (Figure 3B). In water, the average hydrodynamic diameter before modification with GOx was 414 ± 17 nm (pH 5.5); after modification, it increased up to 663 ± 38 nm (pH 6.3). In 9 mM HEPES buffer (pH 7.0), the *d*_h_ of p(AA-BIS) was estimated as 502 ± 17 nm, and after modification, the *d*_*h*_ of p(AA-BIS)-GOx microgels was increased up to 730 ± 67 nm. The buffer concentration also affects the size of the microgels since *d*_h_ first increases and then decreases with an increase in buffer concentration. In pure water, the microgel size is the smallest one, related to the lowered pH. In low buffer concentrations (3 and 9 mM) at pH 7, the microbeads diameter is larger vs water, while for the highest concentration of HEPES (30 mM), a decrease in size was observed. This can be explained as follows: first, at low buffer concentrations, the pH change from 5.5 or 6.3 (for p(AA-BIS) or p(AA-BIS)-GOx, respectively) to 7.0 causes swelling of the microgels due to further deprotonation of carboxylic groups. This process increases the osmotic pressure and repulsion interactions between the ionized carboxylic groups, leading to the observed swelling phenomenon. With a further increase of buffer concentration (30 mM), the increase of ionic strength begins to play a dominant role. The electrostatic repulsion interactions are screened by ions from the solution, and the microgels shrink. This trend is the same for both microgels, with and without the immobilized enzyme. Finally, the sensitivity of p(AA-BIS)-GOx microgels to the presence of glucose was studied. The influence of glucose on the size of the microparticles was determined by DLS measurements after the addition of 25 mM of glucose and waiting over 24 h. The average hydrodynamic diameters of p(AA-BIS)-GOx decreased in all solutions. In water, it dropped down to 423 ± 32 nm, and to 513 ± 22 nm, 534 ± 37 nm, and 676 ± 28 nm in 3, 9, and 30 mM HEPES buffer, respectively (Figure 3B–F). This followed the change in pH, which after 1 day in the presence of glucose dropped to 3.7, 4.1, 4.8, and 6.9 in water, and 3, 9, and 30 mM HEPES, respectively.

**Figure 4:**
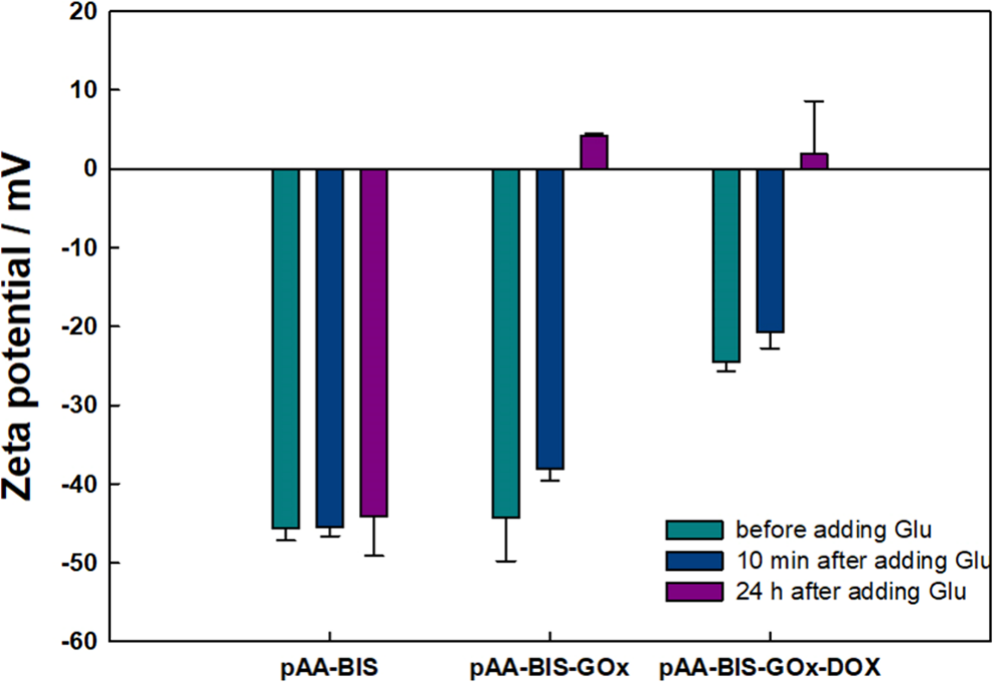
ζ Potential of p(AA-BIS), p(AA-BIS)-GOx, and p(AA-BIS)-GOx-DOX in conditions: before, 10 min after, and 24 h after adding glucose (*c* = 25 mM glucose (Glu)).

The p(AA-BIS)-GOx particles serve as microreactors. Upon the addition of glucose, the enzymatic reaction catalyzed by glucose oxidase starts to occur, resulting in the formation of gluconic acid and hydrogen peroxide. Consequently, the enzymatic activity induces acidification of the solution surrounding the particles, with the acidification subsequently propagating into the bulk over time. The p*K*_a_ of poly(acrylic acid) is approximately 4.8.^40^ As the pH decreases from 7 to lower values, the carboxylic groups of the polymer chains undergo protonation. This protonation facilitates the formation of hydrogen bonds between carboxylic groups, accompanied by a reduction in osmotic pressure, ultimately causing the microgels to shrink. This shrinkage manifests as a decrease in the hydrodynamic diameter of the microgels, a phenomenon confirmed by pH measurements of the solutions after 24 h. To confirm this statement, ζ potential measurements were performed for p(AA-BIS), p(AA-BIS)-GOx, and p(AA-BIS)-GOx-DOX under conditions before adding glucose, 10 min after adding glucose, and 24 h later (Figure 4).

As is evident, the unmodified hydrogel has almost an unaffected surface charge in the absence and the presence of glucose. After modifying the microgel with the enzyme, the addition of glucose results in a significant increase in ζ potential. Within 10 min, the ζ potential increased from approximately −44 mV to −38 mV, and subsequently, after 42 h, it reached 4 mV (Figure 4). This is consistence with DLS measurements and confirms that enzyme-produced acid protonates the carboxyl groups of the polymer chains, and also affects the charge of the attached enzyme, which isoelectric point is about 4.2.^41^ The positive value of the ζ potential suggests that the pH decreases below 4.2.

The same experiment was conducted with doxorubicin-loaded microgel, and a similar trend was observed. However, their initial ζ potential value was less negative. Doxorubicin can interact not only with the ionized carboxylic groups within the polymeric network of p(AA-BIS) but also with the negative charges on the surface of the enzyme located on the modified microgel p(AA-BIS)-GOx. The interaction of doxorubicin with the microgel causes an increase in the ζ potential of the loaded microgel, due to binding drug not only inside but also on the surface of particles.

### 4.3 Enzymatically Triggered Drug Release from Functionalized Microgels

A possibility of controlled release of DOX by adding glucose to a solution of p(AA-BIS)-GOx-DOX microgels loaded with the drug was investigated. Initially, the loading of the microgel with doxorubicin was performed in a solution at pH 7 overnight. These conditions facilitate optimal drug loading, as the carboxylic groups of the polymer chains are primarily deprotonated (the p*K*_a_ of poly(acrylic acid) is approximately 4.8), while the amine groups of doxorubicin are almost fully protonated (the p*K*_a_ of the amine group of doxorubicin is 9.9). Under these conditions, electrostatic interactions between the polymer network and the drug support the drug loading and retention.

**Figure 5:**
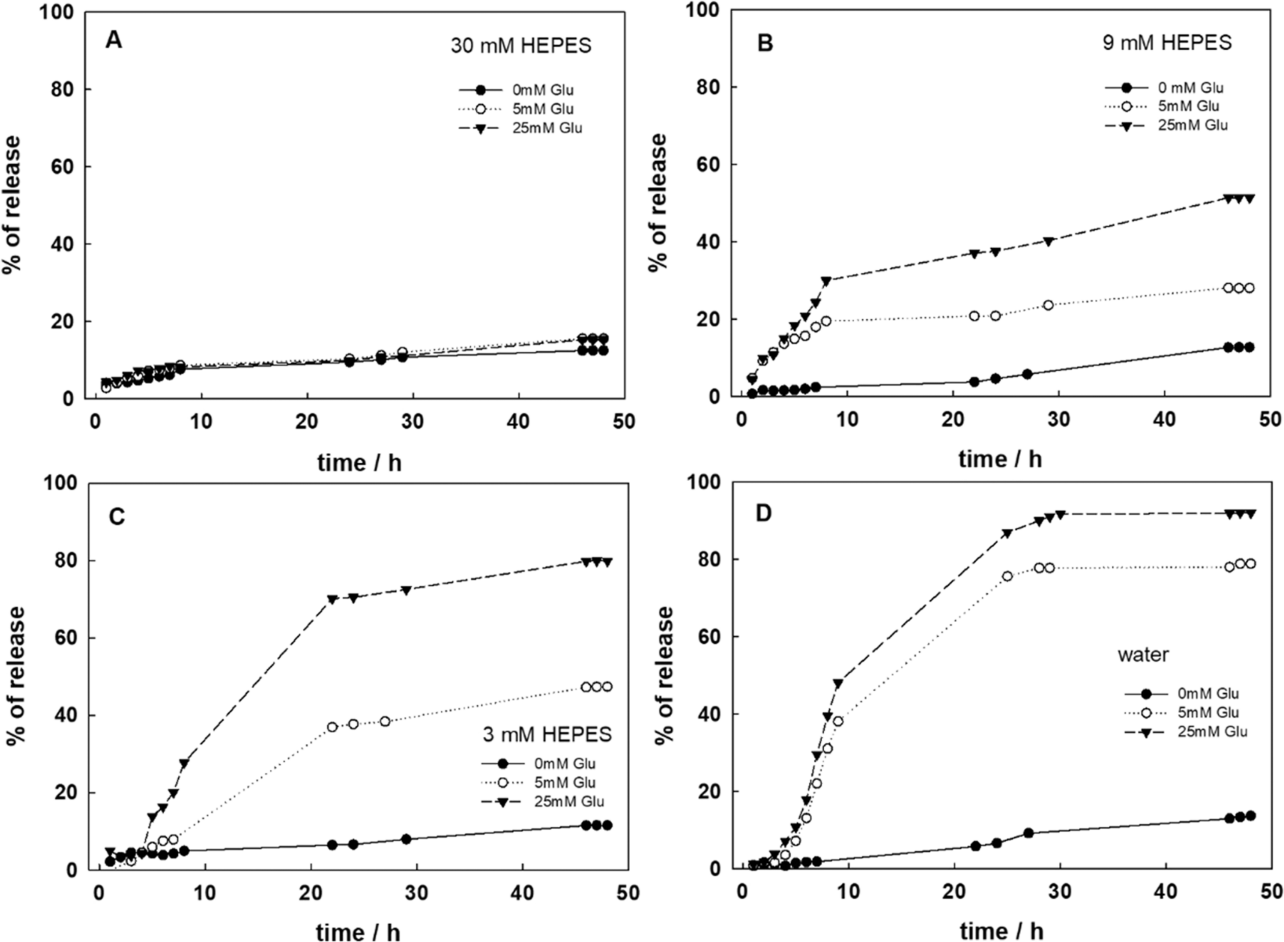
Release profiles for p(AA-BIS)-GOx-DOX at different concentrations of HEPES buffer, pH 7: (A) 30 mM, (B) 9 mM, (C) 3 mM, and (D) water. The glucose concentrations were 0 mM (control sample), 5 and 25 mM.

The drug release experiments have lasted 48 h. The release of doxorubicin was investigated under various conditions: in buffer concentrations of 0, 3, 9, and 30 mM, and with different glucose concentrations as triggers −5 and 25 mM, simulating conditions in healthy and cancer environments, respectively. Control samples were also prepared without glucose addition. The obtained release profiles are shown in Figure 5.

The objective of this task was to trigger the release of the drug through the enzymatic reaction of glucose oxidation. In the presence of glucose, gluconic acid is produced, resulting in a decrease in pH. Due to the predominantly electrostatic nature of the interaction between the matrix and the drug, the release can be triggered by both acidification and alkalization of the solution, as investigated in detail in the literature.^37^ The pH drop causes the protonation of carboxylic groups, resulting in the loss of electrostatic interaction between the microgel and the drug. Consequently, doxorubicin is no longer attracted to the polymer network and can be released from the microgels. This is schematically presented in Figure 1. The crucial factor triggering the release is the presence of glucose at a certain concentration. The use of glucose oxidase is justified because the interstitial glucose concentration in a tumor ranges from ∼15 to 50 mM and the normal range for fasting blood glucose levels is about 5.5 mM. As observed, the release is suppressed in a solution with the highest buffer capacity, regardless of the glucose concentration, as shown in Figure 5A. In this case, where the ionic strength is the highest, leakage related to the weakening of electrostatic interaction was not significant, with less than 8% release efficacy after 48 h.

The decrease in buffer capacity allowed for the enzymatic reaction’s effect to be observed. The activity of the enzyme in p(AA-BIS)-GOx-DOX was calculated as 0.85 U/mL. The amount of generated acid depends on glucose concentrations. Therefore, in the presence of 25 mM glucose, more acid is produced than with 5 mM glucose. This difference can be magnified by buffer concentration, as shown in Figure 5. For buffer concentrations of 9, 3, and water, a difference in the percentage of released drug relative to glucose concentration was observed (Figure 5B–D). It should be noted that the release experiment conducted in water without glucose was performed under the same conditions used for loading DOX, during which almost the entire amount of the drug was incorporated into the microgel. In the release experiment, a very small leakage was observed after 5 h, which increased to almost 10% after 48 h. This leakage could be related to environmental factors, such as slight acidification of the water over time due to carbon dioxide from the air, or potential imperfections in the measuring system. Under these conditions, DOX shows negligible leakage, especially in comparison to its behavior in the presence of glucose.

**Figure 6:**
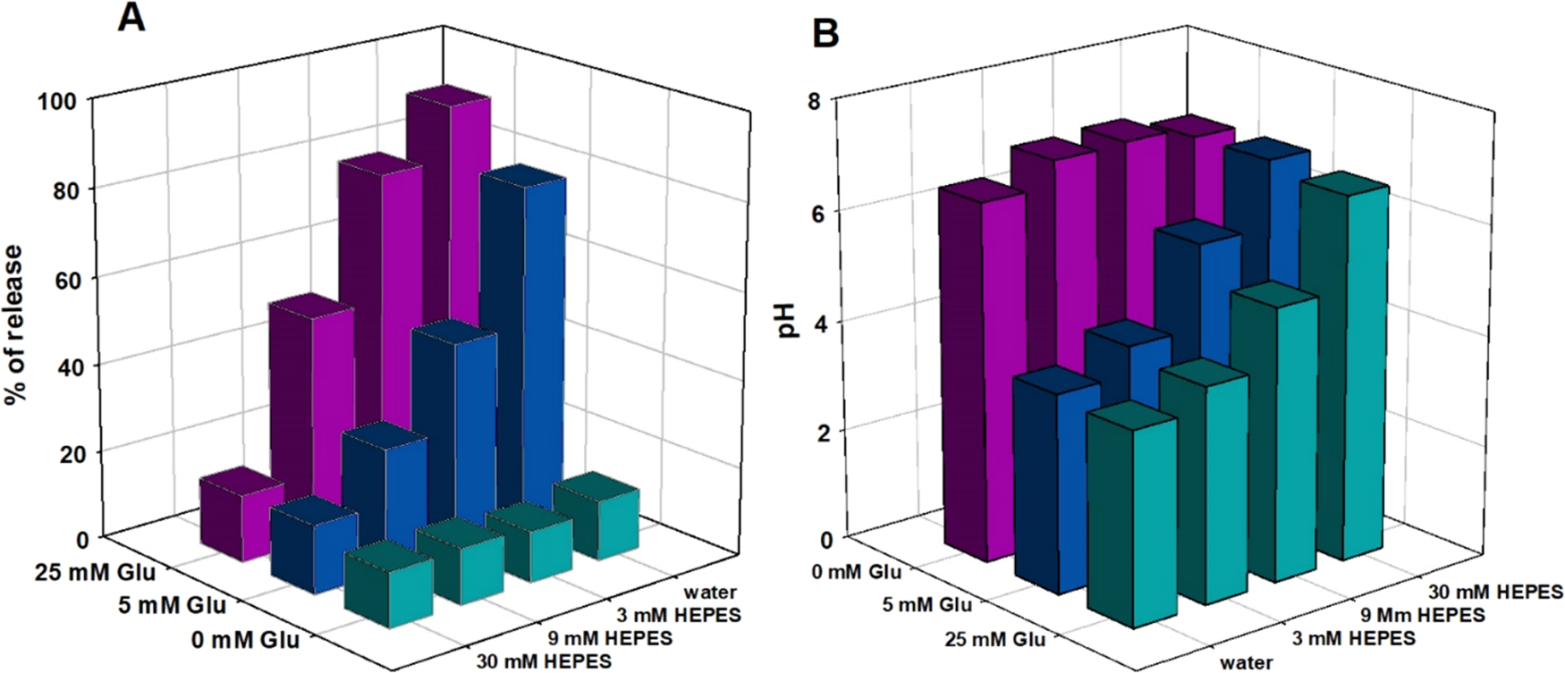
(A) Release percentage for p(AA-BIS)-GOx-DOX at different concentrations of HEPES and glucose after 48 h. (B) pH changes in microgel solutions after 48 h of release experiments under various concentrations of buffer and glucose.

The difference between the amounts of released drug in the presence of 5 and 25 mM of glucose was the highest at 3 and 9 mM HEPES. Under these conditions, 80 and 52% of DOX were released with 25 mM glucose, while for 5 mM glucose, 47 and 28% of DOX were released, respectively. Figure 6A clearly demonstrates that a higher concentration of the enzyme’s substrate (glucose) results in more drug amount being released. Simultaneously, the percentage of released DOX is the highest in water and decreases with increasing buffer capacity for both glucose concentrations. These results demonstrate the potential of the p(AA-BIS)-GOx-DOX microgel for enhanced release of DOX in tumors compared to the bloodstream, where the microgel can accumulate using the EPR effect (see Figure 1).

Observed differences in drug release at various concentrations of buffer and glucose are associated with pH changes generated by the enzymatic reaction. pH alterations occur in the vicinity of the microgel, propagate into the bulk solution, and ultimately reach the values presented in Figure 6B after 48 h. The results depicted in Figure 6A are consistent with the pH values of the solutions presented in Figure 6B.

The kinetics of doxorubicin release from p(AA-BIS)-GOx-DOX microgels under various conditions were also investigated using the zero-order model, first-order model, Higuchi model, and Korsmeyer-Peppas model (see detailed description in Supporting Information (SI)). The Korsmeyer-Peppas model, which describes drug release from polymeric/gel matrices, provided the best fit to the experimental data for glucose-triggered DOX release (see SI Table 1).

## 5 Conclusions

In this study, we have presented the concept of a gel microreactor designed to trigger drug release through an enzymatically generated pH change in the presence of the target substrate. It has been demonstrated the synthesis, characterization, and glucose-triggered drug release behavior of poly(acrylic acid) microgels modified with GOx. The microgel was synthesized using distillation polymerization and exhibited high monodispersity and a uniform spherical shape. The enzymatic modification with GOx introduced a functional layer capable of catalyzing glucose oxidation, leading to localized pH changes and subsequent drug release. The synthesized microgel was well-defined, monodispersed spherical particles. SEM and TEM transmission electron microscopies confirmed the uniform shape and size of both unmodified and enzyme-modified microgels. The enzyme modification led to an increase in particle size, indicating successful GOx immobilization. DLS measurements revealed that the hydrodynamic diameter of the microgels decreased with decreasing pH value due to the protonation of carboxylic groups, causing microgel shrinkage. The enzyme-functionalized microgels exhibited sensitivity to glucose, with the addition of glucose leading to a decrease in hydrodynamic diameter.

The enzyme-functionalized microgel was successfully loaded with doxorubicin with 15% efficiency. The enzymatically triggered drug release experiments demonstrated that doxorubicin release was dependent on glucose concentration and buffer capacity. The highest difference in glucose-triggered release was observed for 3 mM and 9 mM concentrations of HEPES buffer. The study highlighted the potential of these microgels for drug delivery applications utilizing the EPR effect, where drug release is controlled by enzymatically generated pH change. Note that the presented DDS is not limited to the use of glucose oxidase: by modifying the microgel with another enzyme the triggering substrate can be altered, provided that the enzymatic reaction leads to a pH change.

## Supporting Information Available

EDS spectra and TEM image of the microgel, FTIR spectra of the microgel, and fitting kinetic model to drug release for the microgel (PDF)
