# Supplementary material for: Enzymatically Triggered Drug Release from Microgels Controlled by Glucose Concentration
Source: ACS Biomater Sci Eng. 2024 Oct 2;10(10):6415–24. doi: 10.1021/acsbiomaterials.4c01721 (PMC11480938; doi:10.1021/acsbiomaterials.4c01721)
Supplement: Supplementary file 1 — ab4c01721_si_001.pdf [file ab4c01721_si_001.pdf]

Supplementary Informations

Enzymatically Triggered Drug Release from Microgels Controlled by Glucose Concentration

Klaudia Kaniewska[a,b,*], Marcin Mackiewicz[b], Oleh Smutok[c], Mykhailo Gonchar[d], Evgeny Katz[c,*], Marcin Karbarz[a,b]

[a]*Faculty of Chemistry, University of Warsaw, 1 Pasteura, Warsaw, PL 02-093, Poland*

[b]*Biological and Chemical Research Center, University of Warsaw, 101 Żwirki i Wigury Av., Warsaw, PL 02-089, Poland*

[c]*Department of Chemistry and Biomolecular Science, Clarkson University, Potsdam, 13699, NY, United States*

[d]*Institute of Cell Biology, National Academy of Sciences of Ukraine, Lviv, 79005, Ukraine*

**corresponding authors: kkaniewska@chem.uw.edu.pl; ekatz@clarkson.edu*

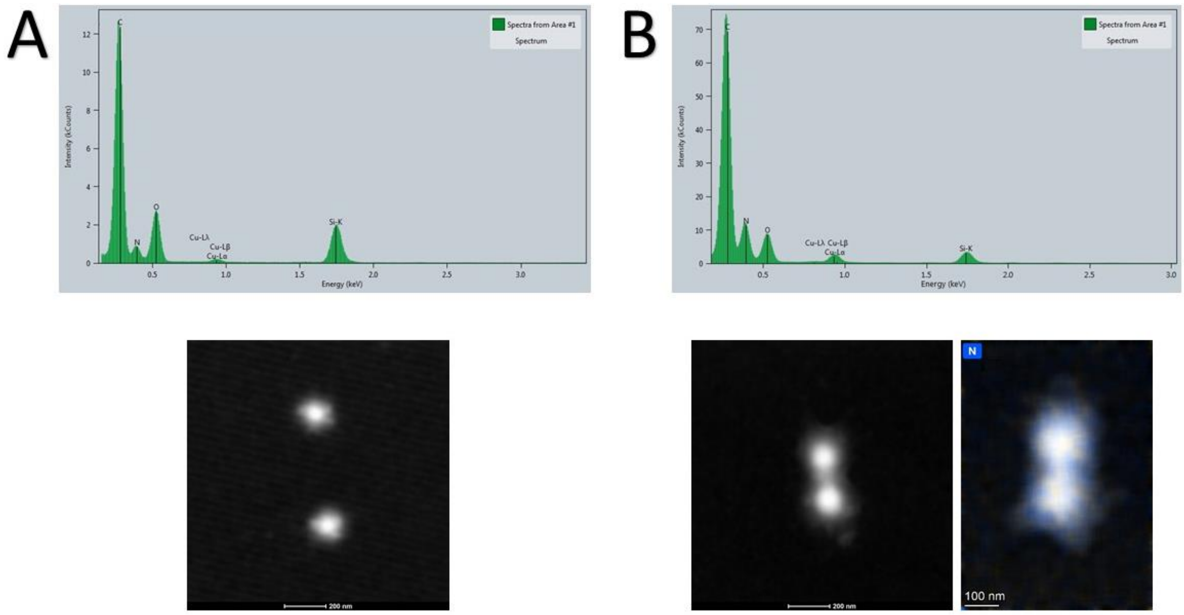

Fig 1s. (A) EDS spectra and TEM image of p(AA-BIS) microgel, (B) EDS spectra, TEM image, and EDS-HAADF elemental mapping of nitrogen of p(AA-BIS)-GOx microgel.

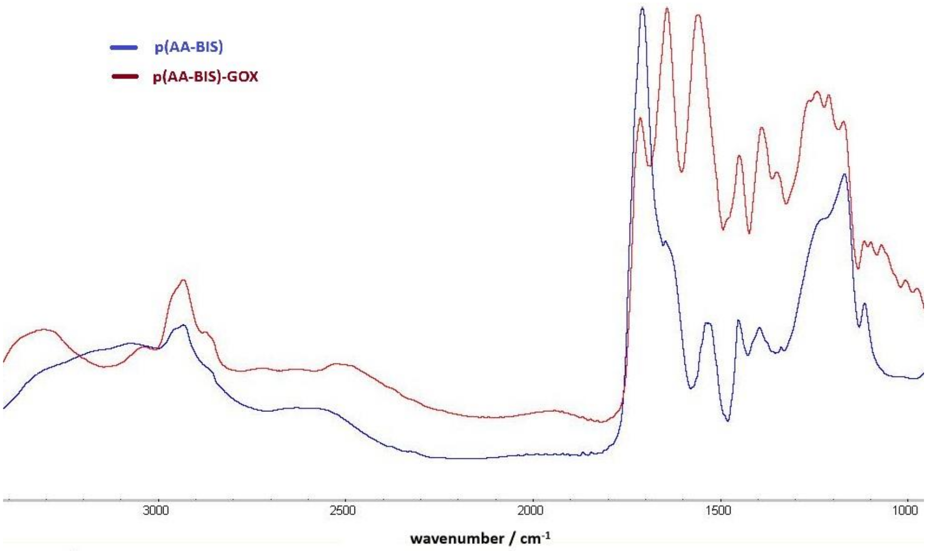

Fig 2s. FTIR spectra of the microgel before and after modification with glucose oxidase.

Fitting kinetics model to drug release data obtained for microgel p(AA-BIS)-GOx-DOX

The described below kinetic models were selected to evaluate release of doxorubicine from p(AA-BIS)-GOx-DOX microgel.[1-4]

1. Zero order model. This model describes concentration independent release. For this model the equation representing doxorubicin release is as follows:

$Q_t = Q_o + K_o\, t$

where Q_t is the amount of drug released at time t, Q_o is the initial amount of drug in t =0, K_o is the zero-order rate constant. The data obtained during release experiments were plotted as percentage of the released drug versus time.

2. First order model. This model describes concentration dependent release. For this model the equation representing doxorubicin release is as follows:

$logC_t = logC_o - K\, t/2.303$

where C_t is the percent of drug remaining at time t, C_o is the initial concentration of the drug, K is the first order rate constant. The data obtained during release experiments were plotted as log of percentage of the remaining drug versus time.

3. Simplified Higuchi model. This is the classical model for drug delivery release kinetic studies with Fickian diffusion of drug from insoluble matrix. This model assumes among other that matrix does not swell or dissolute during release, despite this, due to its simplicity it is often used to study kinetics of release from hydrogel matrices. For this model the equation representing doxorubicin release is as follows:

$Q_t = K_H\, t^{1/2}$

where Q_t is the amount of drug released at time t, K_H is the Higuchi release kinetic constant. The data obtained during release experiments were plotted as percentage of the released drug versus time.

4. Korsmeyer-Peppas model. This kinetic model describes drug release from polymeric/hydrogel matrixes. This model allow to model kinetics for both swellable and un-swellable matrixes, and allow to determine mechanism of release based on parameter n. For this model the equation representing doxorubicin release is as follows:

$log\,(Q_t / Q_\infty) = log\,K + n log\,t$

where Q_t is the amount of drug released in time t, Q_∞ is the amount of drug released after time ∞, n is drug release exponent, K is release rate constant. The data obtained during release experiments were plotted as log of percentage of the released drug versus log of time. The data from first 60% of release are taken for modelling. The value of parameter n for spherical system equal 0.43 points to Fickian diffusion release mechanism, for n between 0.43 and 0.85 the anomalous (non-Fickian) transport takes place, when n=0.85 it indicates the case II transport (zero order release), and $n > 0.85$ correspond to the super case II transport.

Table 1. presents the coefficient of determination (R^2) of zero-order, first-order, Higuchi and Korsmeyer–Peppas models fitted to release data.

Table 1S. The coefficient of determination (R^2) values obtained from selected models for modelling the doxorubicin release kinetic in different conditions for first 60% of release.

		**5 mM**	**25 mM**
**0 mM**	Zero Order	0.8798	0.9086
	First Order	0.8532	0.8706
	Higuchi	0.7772	0.8151
	Korsmeyer-Peppas	**0.8889**	**0.9518**
**3 mM***	Zero Order	0.9545	0.9359
	First Order	0.9570	0.9238
	Higuchi	0.9748	0.9184
	Korsmeyer-Peppas	0.9451	0.9504
**9 mM**	Zero Order	0.9428	**0.9855**
	First Order	0.9518	0.9790
	Higuchi	**0.9831**	0.9605
	Korsmeyer-Peppas	0.9708 n=0.67	**0.9828** n=0.86
**30 mM**	Zero Order	0.9591	0.9067
	First Order	0.9620	0.9085
	Higuchi	0.9888	0.9470
	Korsmeyer-Peppas	**0.9911**	**0.9520**

** the models were fitted for smaller number of points.*

1 Siepmann, J.; Siepmann, F. Modeling of diffusion controlled drug delivery, J Control. Rel. 2012, 161, 351-362

2 Ritger, P.L.; Peppas N.A. A Simple Equation For Description Of Solute Release I. Fickian and Non-Fickian Release from Non-Swellable Devices in The Form of Slabs, Spheres, Cylinders Or Discs, J. Control. Rel. 1987, 5, 23-36.

3 Ritger, P.L.; Peppas N.A. A Simple Equation for Description Of Solute Release II. Fickian and Anomalous Release from Swellable Devices J. Control. Rel. 1987, 5, 37-42.

4 Dash, S.; Murthy, P.N.; Nath, L.; Chowdhury, P. Kinetic Modeling On Drug Release From Controlled Drug Delivery Systems, Acta Pol. Pharm. 2010, 6, 217-223.
